# Effect of Microfiltration Membrane Configuration in Microplastics Recovery from Wastewater Treatment Effluent

**DOI:** 10.3390/membranes15050137

**Published:** 2025-05-02

**Authors:** Rubén Rodríguez-Alegre, Sergi Durán-Videra, Laura Pérez Megías, Montserrat Pérez-Moya, Julia García-Montaño, Carlos Andecochea Saiz, Xialei You

**Affiliations:** 1 Leitat Technological Center, Circular Economy & Decarbonization Department, C/de la Innovació 2, 08225 Terrassa, Spain; sduran@leitat.org (S.D.-V.); rrodriguez@leitat.org (R.R.-A.); lperez@leitat.org (L.P.M.); candecochea@leitat.org (C.A.S.); jgarcia@leitat.org (J.G.-M.); 2 Chemical Engineering Department, Universitat Politècnica de Catalunya, EEBE, C/Eduard Maristany 10-14, Campus Diagonal-Besòs, 08019 Barcelona, Spain; montserrat.perez-moya@upc.edu

**Keywords:** microplastics, microfiltration, isolation, quantification, recovery, circular economy

## Abstract

Water scarcity has driven the use of wastewater treatment plant (WWTP) effluents as reclaimed water, highlighting the need to overcome challenges such as the presence of emerging contaminants, particularly microplastics (MPs), which WWTPs are unable to effectively remove. Membrane-based processes, such as microfiltration, have demonstrated high efficiency in the removal of suspended solids, and their application for MP removal is currently under investigation. This study assesses the influence of microfiltration membrane spacer size (1 mil and 80 mil) and geometry—diamond and corrugated—on MP recovery performance, using synthetic wastewaters with varying MPs concentrations. The results indicate the superior performance of large corrugated and small diamond-shaped membranes, as both exhibited the highest and comparable permeate flux, with no MP retention within the membrane element. All microfiltration membranes achieved an 80% recovery of the influent as safe reclaimed water and demonstrated an MP recovery efficiency exceeding 99%, with 100% rejection for fragments and up to 98% rejection for fibres.

## 1. Introduction

Water scarcity is an increasingly pressing global concern, and the Mediterranean area is facing more severe and frequent drought episodes, affecting both rural and urban areas. In this context, reusing wastewater effluent as reclaimed water is not just an option but a necessity to ensure a sustainable water supply. However, water reuse also introduces new challenges, particularly the presence of contaminants of emerging concern, such as microplastics (MPs), which can pose significant risks to both human health and ecosystems.

In response to these challenges, the European Reused Water Regulation 2020/741 included contaminants such as MPs in the list of substances to be monitored, anticipating their inclusion in the list of regulated substances in the near future. Adopting treatment technologies to meet the current regulations and anticipate future scenarios is therefore crucial for producing high-quality reclaimed water that could be potentially used as an influent of drinking water treatment plants, as is already being practised in some places due to extreme droughts.

Plastics are omnipresent in human activities, and a significant portion ends up in water bodies, either through direct discharge or as wastewater treatment plant (WWTP) effluent discharge [1]. The presence of MPs in wastewater originates from a variety of sources, with washing machines being the primary contributors [2], releasing fibres and fragments from clothing that ultimately reach municipal channels and, eventually, water bodies [3]. MP pollution in water is an environmentally significant issue that also generates substantial social concern, capturing the attention of scientists and policymakers alike [4]. These tiny plastic particles, with sizes ranging between 5 mm and 1 µm, pose a high potential risk to human health, being linked to organ dysfunction, immune response issues, and DNA damage, as well as environmental harm, such as risks to biodiversity and the adsorption/release of toxic compounds, among others [5,6].

Numerous studies have been conducted on the removal and degradation of MPs in liquid matrices, focusing on water treatment. However, the recovery of MPs as added-value products is still unexplored. Conventional methods such as coagulation [7,8] and membrane bioreactors [9] are especially interesting for their simplicity, although they often result in a mixture of MPs and sludge, which is sometimes discharged into soil, transferring the problem from the aquatic environment to the terrestrial environment [10].

In contrast, other technologies, such as advanced oxidation processes (i.e., photocatalysis and UVC/H_2_O_2_), are aimed at degrading MPs and their leachates [11,12]. However, these technologies require extended time periods to be effective as an incomplete oxidation could lead to the formation of hazardous by-products [13].

In addition to previous technologies, which were developed for MP removal or degradation, recent studies have explored MP recovery from water streams to give them a second life within the circular economy concept. This approach views MPs as an added-value product, helping to reduce their environmental impact [14]. Advanced treatments such as electrocoagulation [15] and flotation using microbubbles [16] enable the recovery of MPs. However, electrocoagulation is an energy-intensive process while flotation may be less efficient due to microbubble size requirements [17,18].

Historically, pressure-driven membrane processes have proven effective in contaminant concentration [19]. These processes are gaining attention for their potential in innovative industrial applications, such as the use of ultrafiltration [20] and microfiltration (MF) [20,21,22,23] for the recovery of MPs from waste streams. MF is a particularly promising technology due to the size of MPs falling within the effective range of MF membranes, enabling high water flow rates and large-volume treatment with reduced energy consumption [24]. However, for the optimal functionality of the membranes, some structural parameters need to be assessed such as the spacer configuration (i.e., geometry and size).

The importance of spacers in membrane filtration, including both spiral-wound and flat-sheet membranes, has been recognised for decades. Previous lab-scale works have compared different spacer geometries, such as parallel and diamond, noting their similar operational performance in wastewater treatment for solid removal [25]. Diamond-shaped spacers, which promote crossflow, leading to reduced membrane fouling, are the most cost-effective option in terms of manufacturing [26] and are extensively used in water treatment processes with low solid concentrations due to the presence of obstruction points in the pattern. By contrast, corrugated spacers demonstrate higher efficiencies when treating water with a high solid content, due to their lower pressure drop compared to the diamond shape.

However, limited information is available regarding how spacer configuration affects MP retention [27], as their morphology could significantly affect filtration behaviour. Fragments act as rigid particles and are retained by size exclusion, while fibres may align with the flow, crossing the membrane pores or being entangled within the spacers’ structure. Therefore, there is a knowledge gap regarding the effect of membrane element spacer size and geometry for the minimisation of membrane permanent fouling due to particle embedding while maximising fibre rejection.

This study aims to assess the effectiveness of MF membranes with representative spacer sizes and geometries for the recovery of MPs from synthetic wastewater. In this work, two spacers of 80 mils will be compared with diamond and corrugated geometries on one hand and diamond-shaped spacers with different sizes (31 mil to 80 mil) on the other hand. The results will be analysed using circularity indicators for water treatment and MP recovery, and the performance of the MF process will be compared with the data reported in the existing literature.

## 2. Materials and Methods

### 2.1. Reagents and Equipment

In this study, a pH 3 solution of HNO_3_ (0.001 M) and pH 11 solution of NaOH (0.001 M) were prepared by using pure reagents supplied by Scharlab to clean the membranes after the filtration experiments. FeSO_4_·7H_2_O and H_2_O_2_ 30% supplied by Scharlab were used for water matrix purification in the quantification protocol. NaCl (99%) for MP flotation was supplied by Merck (Rahway, NJ, USA).

**Figure 1:**
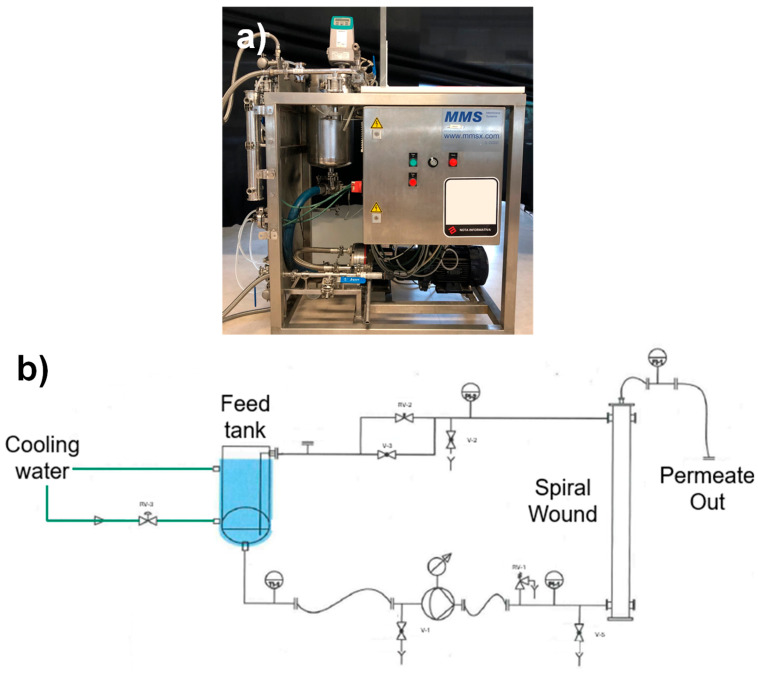
(**a**) Picture and (**b**) flowsheet diagram of SW-18 filtration unit representing batch mode operation (MMSX, Switzerland).

MF membranes with a 0.20 µm pore size (the most representative in the current market) were supplied by Synder (Vacaville, CA, USA) and MANN + HUMMEL (Wiesbaden, Germany). Detailed specifications for the membranes are provided in Table 1. Diamond spacers were selected as they are the most commonly used at the industrial scale, while corrugated spacers are the ones typically applied in the treatment of streams with high solid concentrations—considering MPs as suspended solid—as detailed in previous sections. The membranes were tested using an SW-18 filtration unit (MMSX, Urdorf, Switzerland) operating in crossflow mode under batch conditions with retentate recirculation to assess their filtration performance (Figure 1).

For MP quantification, a first isolation protocol (detailed in Section 2.3) was carried out. After MP isolation, an optical microscope coupled with a UV lamp from Axioplan 2 (Zeiss, Barcelona, Spain) was used to quantify the fluorescent MPs.

The filtrations were performed by using synthetic wastewater generated by washing 1 kg of synthetic polyester garments (with no detergent) with tap water in a domestic washing machine. Three consecutive washing cycles were performed, obtaining synthetic samples of 52,128 ± 38 particles L^−1^ in the first sample; 17,932 ± 1466 particles L^−1^ in the second sample; and 4690 ± 129 particles L^−1^ in the third sample.

### 2.2. Filtration Protocol

The filtration of the synthetic wastewater was conducted in the SW-18 filtration unit by circulating (in batch mode) the samples at 900 L h^−1^ and under a trans-membrane pressure of 1.50 bar, with a recovery rate of 80%. The samples and system were kept at 25 ± 2 °C throughout all experiments.

The membranes were weighed at the beginning and end of the experiments to measure MPs embedded in the membrane’s structure considering that, being a synthetic wastewater, the presence of other pollutants was negligible. To compare their weight before and after filtration, the membrane was submerged in distilled water before and after each filtration and left to drain for 1 h. The drained membranes were then weighed on a balance, calculating the MPs embedded (*MPs_retention_*) in grammes, as shown in the following equation, Equation (1):

(1) $${MPs}_{retention}=m_{final}-m_{initial}$$

where *m_final_* and *m_initial_* are the final and initial membrane weight (g), respectively.

In addition, the collected samples (feed, retentate and permeate) were filtered under vacuum and dried in an oven for 1 h at 105 °C and then weighed.

The measurement of the MPs (*MPs_weighted_*) in grammes for each stream was obtained using Equation (2) as follows:

(2) $${MPs}_{weighted}=m_{filtered\;sample}-m_{filter}$$

where *m_filtered_* is the weight of the filter after sample filtration and drying in the oven (g) and *m_filter_* is the weight of the filter before the sample filtration.

The MP percentual retention efficiency (*R_MPs_*) was calculated based on MP permeation, as is shown in Equation (3):

(3) $$R_{MPs}=\left(1-\frac{{MPs}_{feed}{-MPs}_{permeate}}{{MPs}_{feed}}\right)\times 100$$

where *MPs_feed_* and *MPs_permeate_* are the microplastics concentration (particles L^−1^) in the feed and permeate streams, respectively.

To compare the performance of the different membranes, the relative permeate flux (*J_norm_*) was used, calculated using Equation (4):

(4) $$J_{norm}=\frac{J}{J_{0}}$$

where *J*_0_ and *J* are the permeate flux (LMH) at the beginning and at the end of the filtration experiment.

It is worth noticing that, due to the batch operating mode with retentate recirculation, the liquid level in the feed tank gradually decreases as permeate is collected. At the end of each batch, the remaining liquid in the feed tank is referred to as the retentate. However, this fraction is not used for calculating MP retention. Instead, retention is calculated based on the initial concentration of MPs in the feed and the concentration measured in the accumulated permeate over the course of the batch.

After each filtration, the membranes were cleaned following the next protocol: (I) a flush with distilled water to remove superficial fouling; (II) an alkaline cleaning using a pH 12 NaOH solution for 30 min at 40 °C; (III) a flush with distilled water to restore the pH value to neutral (pH 7 ± 0.5); (IV) an acid cleaning with HNO_3_ for 15 min at pH 3 and room temperature; and (V) a final flush to return the pH to neutral (7 ± 0.5).

### 2.3. Microplastics Isolation and Quantification Protocol

**Figure 2:**
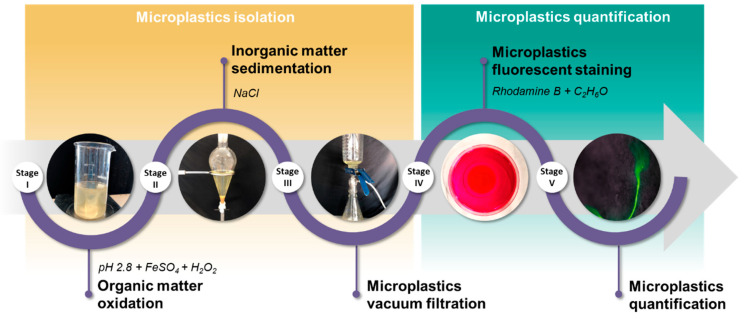
MP isolation protocol followed in this study.

A customised protocol was developed for the effective isolation of MPs. Building on previous studies, different methodologies were integrated to account for the complexity of real samples and to fully leverage the capabilities of the available technologies (Fenton, density separation, vacuum filtration, and fluorescent staining) [28] (Figure 2).

The Fenton process was used for the oxidation of organic matter. The Fenton reagent, FeSO_4_·7H_2_O and H_2_O_2_, was added to the wastewater sample in proper amounts for the COD content to generate hydroxyl radicals (•OH) to breakdown the organic matter attached to the MPs [29,30].

The oxidised sample was saturated with 120 g L^−1^ of NaCl to increase the matrix density and precipitate inorganic materials while facilitating MP flotation, minimising interference during subsequent filtration [31,32]. Vacuum filtration using glass fibre filters with a pore size of 1.2 µm was used for MP retention from the supernatant.

The filter containing MPs was submerged in a staining solution of 200 mg L^−1^ rhodamine B in ethanol [33] to enhance the visualisation and identification of MPs under fluorescence microscopy.

The stained filters were examined with an optical microscope equipped with a UV lamp at a wavelength of 567 nm. A thorough examination of the filter was performed to quantify the fluorescent particles with a size between 5 mm and 1.2 µm according to the most extended definition of MPs’ size range (5 mm to 1 µm) [34]. Each sample was analysed in duplicate to obtain a more representative measure.

### 2.4. Water Recovery Monitoring Indicator

To evaluate the treatment process’s efficiency, several indicators were assessed within the framework of a circular economy. These indicators were not only related to MP recovery but also to water recovery.

The overall recovery of water (in %) potentially available for reuse, according to Spanish legislation (Royal Decree 1620/2007), was evaluated following Equation (4) [35]:

(5) $$W_{rec}=\frac{V_{tw}}{V_{is}}\cdot 100$$

where *W_rec_* is the recovered water indicator in %, and *V_tw_* (L) and *V_is_* (L) are the volumes of reclaimed water and of the initial sample, respectively.

## 3. Results and Discussion

### 3.1. Membrane Assessment

**Figure 3:**
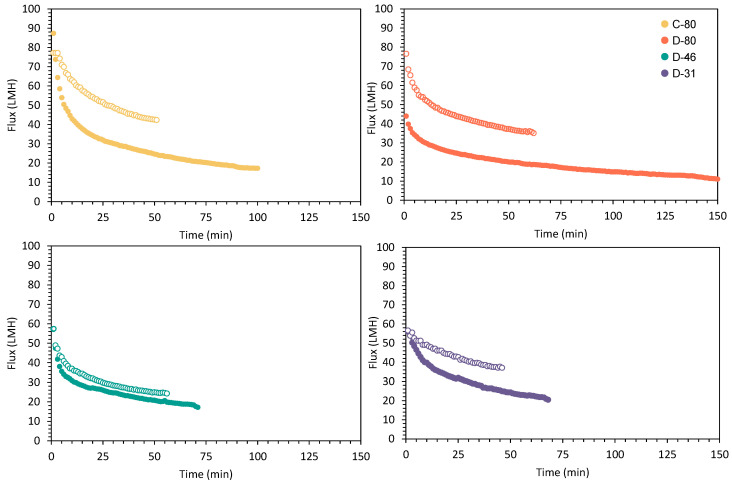
Flux comparison between the first synthetic sample as filled dots (52,128 ± 3836 MPs L^−1^) and the second synthetic sample as empty dots (17,932 ± 1466 MPs L^−1^).

Comparing the filtration performance by using two different synthetic samples (with 52,128 ± 3836 and 17,932 ± 1466 particles L^−1^), a significantly higher permeate flux in all four membranes when treating the second synthetic sample can be explained by its lower initial MP concentration. In Figure 3, the flux evolutions of each experiment can be observed.

When comparing membranes with the same spacer geometry (diamond), D-46 showed the lowest permeate fluxes, starting at 57.56 ± 0.95 LMH for both samples and being reduced to 17.24 LMH (0.30 LMH of relative permeate flux) for the first synthetic sample and to 24.32 LMH (0.42 LMH of relative permeate flux) in the second synthetic sample. In addition, this membrane showed high retention rates of microplastics in its structure, finding a similar behaviour as D-80. In contrast, D-31 showed a lower flux reduction throughout the experimental period (0.42 and 0.77 LMH of relative permeate flux for the first and the second synthetic samples, respectively) (Figure 3).

This phenomenon has been previously reported, as larger spacer sizes tend to increase membrane fouling in similar flux conditions since they reduce the linear velocity within the membrane element, while smaller spacer sizes show improved performance in mitigating the flux decrease rate [36].

When comparing the diamond and corrugated spacers geometries with the same size (C-80 and D-80), the C-80 membrane showed the highest initial permeate fluxes, with 87.33 ± 0.48 LMH for the first synthetic sample and 77.19 ± 3.85 LMH for the second synthetic sample; they were reduced to 17.27 ± 0.86 LMH (0.20 LMH of relative permeate flux) in the first filtration and 42.37 ± 3.81 LMH (0.55 LMH of relative permeate flux) in the second filtration by the end of the tests. In contrast, D-80 showed lower initial fluxes (44.02 ± 3.96 and 76.61 ± 2.30 LMH for the first and second synthetic samples, respectively), finding a decrease to 10.34 and 35.12 LMH (0.23 and 0.46 LMH of relative permeate flux).

**Figure 4:**
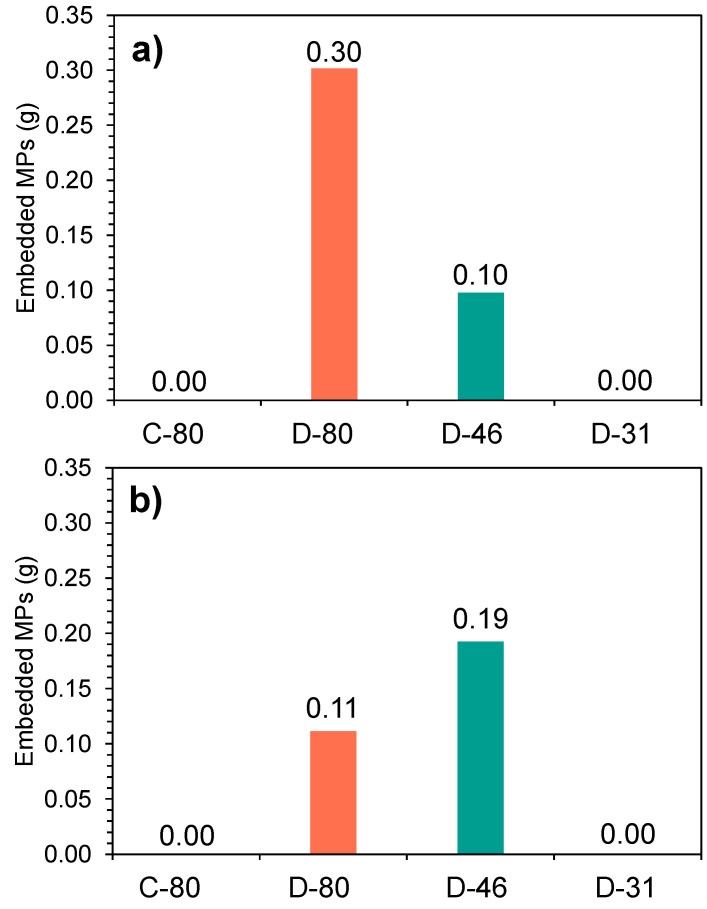
MP concentration retained in the membrane for (**a**) the first synthetic sample (52,128 ± 3836 MPs L^−1^) and (**b**) the second synthetic sample (17,932 ± 1466 MPs L^−1^).

The D-80 membrane showed a high retention rate of MPs within its membrane structure, with more than 3 g of MPs embedded when the first synthetic sample was filtered, while in C-80, the MP retention in its membrane structure was negligible, as can be seen in Figure 4a,b. This fact can be explained, as the C-80 spacer presents a less tortuous geometry, providing fewer sites where the MPs can become stuck, despite having a comparable linear velocity.

Based on these results, the D-80 and D-46 membranes could be discarded for further experimentation since the MP retention in their structure is a clear handicap when working in MP recovery. In order to validate the selection of the membranes of D-31 and C-80, an additional set of experiments was conducted to test their reliance at steady-state.

Finally, it is worth noting that after each filtration, the membrane elements were cleaned according to the protocol detailed in Section 2.2 to assess the presence of permanent fouling. The obtained results (Figure 3) showed that all membranes recovered their initial flux, concluding that the MPs embedded within the structure in the first filtration did not significantly reduce their membrane filtration capacity.

### 3.2. Membrane Validation

According to the preliminary results shown in the previous section, the D-31 and C-80 membranes were assessed through a third filtration using a synthetic sample with 10 times fewer MPs than the previous ones (4690 ± 129 particles L^−1^) to test the reliability of the previous results. The results confirmed that a steady state was reached, as the reduction in flux was less pronounced than in the previous tests, suggesting the minimal permanent fouling of the membranes.

**Figure 5:**
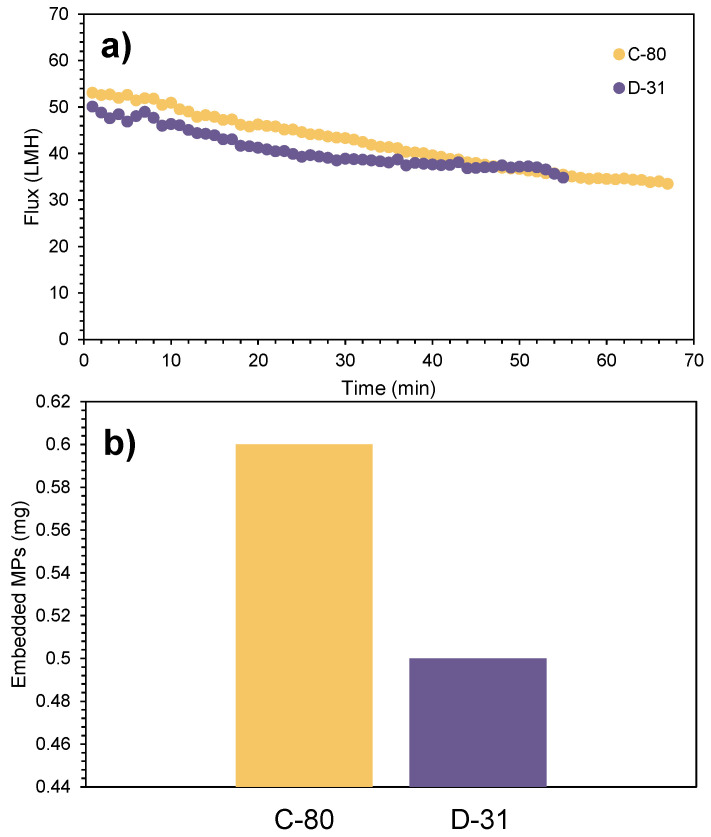
(**a**) Flux comparison between membranes C-80 and D-31 in the third synthetic sample; (**b**) MP concentration retained in the membrane for the third synthetic sample (4690 ± 129 particles L^−1^).

Similar behaviour could be observed in both membranes in terms of permeate flux (Figure 5a), highlighting the good performance of both membranes for MP recovery, despite their distinct characteristics. Regarding MP concentration and membrane fouling, neither membrane demonstrated a significant retention capacity for MPs within their structure (Figure 5b). Specifically, the corrugated structure of C-80 allowed a lower flux drop, minimising MP deposition on the membrane surface, whereas the higher turbulence generated by the smaller size of D-31 also contributed to a lower MP deposition. Therefore, it can be concluded that both membranes are capable of concentrating and recovering MPs effectively.

### 3.3. Microplastics Isolation and Quantification

**Figure 6:**
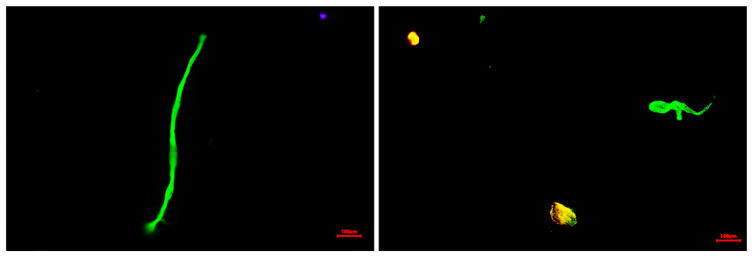
Fragments and fibres isolated following the presented protocol for their quantification.

To gain a more precise understanding of membrane efficiency in terms of retained and potentially recoverable MPs, the isolation protocol described in Section 2.3 was applied and the MPs were quantified in two different fractions: fragments and fibres (Figure 6).

This study on MP particle quantification allows for a more in-depth comprehension of the process itself. The finding of this study (Table 2) demonstrates the viability of using MF to remove MPs from wastewater streams. For those cases where the objective is to treat water to fulfil the current legislation, all membranes tested in this study were able to remove more than 99% of MPs from the wastewater.

MP recovery was calculated considering the MP content in the retentate and permeate streams since the particles embedded in the membrane’s structure could not be recovered for their reuse and were considered permanently fouled.

Although the fragments were totally rejected by all the selected membranes, fibres were detected in the permeate stream in concentrations below 2% in all cases. This finding is in line with the results reported in the literature in which fibres could potentially cross the membrane longitudinally due to their shape [37]. This behaviour is similar when using all the synthetic samples tested in this study, thus showing high replicability.

The study of MP content throughout the isolation protocol allowed the detection of the full removal of MP fragments from the feed stream, but further work is still needed for the effective removal of fibres from the permeate stream.

However, it is worth noting that the removal of fibres is related to the natural entanglement of the fibres increasing the rejection capacity of the membrane. In addition, the electrostatic interaction between the fibres may generate aggregates as well, increasing their rejection.

### 3.4. Circularity Indicator Assessment and Comparative Study

To compare the results of this study with previously published research, Table 3 summarises the recovery of MPs obtained in other studies focused on the treatment of urban wastewater or laundry wastewater. This Table provides an overview of their type of effluent, the technology employed, and the recovery ratio of MPs. The data included in this Table pertinent to the present study are those obtained using C-80 and D-31 membranes, which were selected as the best options.

Besides the data on MP recovery, the water recovery ratio was also assessed in this study. However, it was not included in Table 3 as water recovery was not a specified objective in any of the other summarised studies.

It is important to note that, as of now, there is neither a harmonised definition of MPs nor a standardised MP quantification method. For this reason, the studies showed in Table 3 comprise different MP size ranges and quantification methods, complicating the comparison of their results.

It can be observed that previous studies on membrane filtration reported recovery ratios ranging from 72% to 98%, which are significantly lower than the values obtained within this study (>99%).

The main advantage of using membrane processes is the fact that recovered MPs could potentially be reused as additives in the production of polymer products, contributing to a circular approach to plastic management.

Traditional methods such as coagulation and the use of a membrane bioreactor reported high retention rates but still demonstrated lower efficiencies than those reported in this study [7,8,9]. In addition, these methods often resulted in a mixture of MPs with sludge, requiring additional treatments for effective MP isolation and recovery, reducing overall efficiency and increasing operational costs.

Most of the advanced treatment methods (i.e., electrocoagulation and microbubble flotation) exhibited removal rates lower than those achieved with membrane-based processes [12,15,16]. In addition, they were burdened by higher operational costs due to their substantial energy requirements.

AOPs, such as photocatalysis and UVC/H_2_O_2_, showed low degradation rates even after extended treatment times (more than 2 h), primarily due to the highly recalcitrant nature of MPs [11,12]. While these technologies can be useful for improving biodegradability when combined with biological processes, they incur significant operational costs. Moreover, these oxidative technologies aim to degrade the material, reducing the potential for waste reuse in line with CE principles, ultimately rendering the manufacturing industry less sustainable. In addition, the incomplete oxidation of MPs can lead to the formation of smaller particles with higher potential risks for the environment.

Regarding water recovery, although this study achieved the recovery of 80% of the inlet as safe reclaimed water, MF has the potential to exceed 90%. This discrepancy is attributed to the limitations of the filtration equipment used in this study. This process could potentially achieve higher water recovery ratios when working in continuous mode instead of batch mode.

## 4. Conclusions

The results of this study highlight the efficiency of MF in MP recovery. The feasibility of MF for MP recovery was evaluated by investigating membranes with different spacer sizes and geometries for recovering MPs from synthetic wastewater generated by garment washing.

This study revealed that large, corrugated spacers (C-80) and small, diamond-shaped spacers (D-31) are the most effective configurations for MP removal from wastewater, as they exhibited the lowest reductions in permeate flux and the minimal configuration retentions of MPs within the membrane element. Both membrane configurations demonstrated comparable performance across three filtration cycles although via different mechanisms. Corrugated spacers offer less tortuosity than diamond-shaped ones and, thus, fewer sites for MP deposition, while small spacers offer a higher linear velocity than large ones, impeding deposition. However, a multi-cycling study should be carried out in order to better understand the long-term performance of the selected membranes.

Additionally, an isolation protocol for MP quantification was implemented, integrating physical separation, chemical oxidation, and the fluorescence capacity of rhodamine B. This protocol enabled a comprehensive assessment of MP rejection, demonstrating a 100% removal rate for MP fragments, while up to 2% of microfibers were able to pass through the membrane. These findings underscore the need for further research in terms of MP permeation and membrane fouling depending on the material of the MPs and membrane spacer characteristics to enhance microfiber removal from wastewater streams.

Finally, the assessment of circularity indicators demonstrated the superior performance of MF compared with other technologies in terms of both MP and water recovery. These results highlight the potential of membrane-based processes (particularly MF) for the recovery of MPs as a secondary raw material, contributing to a more sustainable approach to wastewater treatment.

## Figures and Tables

**Table 1:** Specification for each MF membrane used in this study.

Parameter	Unit	V0.2-5CB-1812F	V0.2-5B-1812F	V0.2-3B-1812F	Turboclean 1812-MV020-31
Code	-	C-80	D-80	D-46	D-31
Provider	-	Synder	Synder	Synder	MANN + HUMMEL
Membrane material	-	PVDF	PVDF	PVDF	PVDF
Spacer material	-	PP	PP	PP	PP
Membrane area	m^2^	0.167 (1.80 ft^2^)	0.167 (1.80 ft^2^)	0.269 (2.90 ft^2^)	0.230 (2.50 ft^2^)
Spacer size	Mil	80	80	46	31
Pore size	µm	0.20	0.20	0.20	0.20
Max temperature	°C	55 (131°F)	55 (131 °F)	55 (131 °F)	50 (122 °F)
Max pressure	bar	8.30 (120 psi)	8.30 (120 psi)	8.30 (120 psi)	10.00 (145 psi)
pH range	-	2–10	2–10	2–10	2–10
Spacer type	-	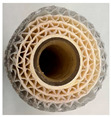 Corrugated	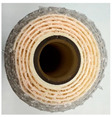 Diamond	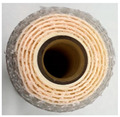 Diamond	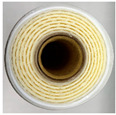 Diamond

**Table 2:** MP count for each sample taken from membrane assessments.

Parameter		Unit	First Synthetic Sample				Second Synthetic Sample				Third Synthetic Sample	
			C-80	D-80	D-46	D-31	C-80	D-80	D-46	D-31	C-80	D-31
Feed	Fragment	Particles L^−1^	41,004 ± 3280				12,446 ± 1247				2370 ± 47	
	Fibre	Particles L^−1^	11,124 ± 556				5486 ± 219				2319 ± 82	
Retentate	Fragment	Particles L^−1^	56,082 ± 2243	58,162 ± 4653	61,204 ± 42,384	62,961 ± 37,778	22,969 ± 411	20,569 ± 1850	24,084 ± 481	25,635 ± 256	9821 ± 687	12,346 ± 370
	Fibre	Particles L^−1^	11,525 ± 1037	9129 ± 730	10,961 ± 219	12,562 ± 126	3683 ± 64	3222 ± 320	4244 ± 424	4803 ± 48	1729 ± 172	2829 ± 169
Permeate	Fragment	Particles L^−1^	0 ± 0	0 ± 0	0 ± 0	0 ± 0	0 ± 0	0 ± 0	0 ± 0	0 ± 0	0 ± 0	0 ± 0
	Fibre	Particles L^−1^	88 ± 3	83 ± 7	92 ± 8	78 ± 2	73 ± 3	70 ± 4	82 ± 4	102 ± 6	72 ± 7	34 ± 0
MP recovery *		%	99.9	99.9	99.9	99.9	99.7	99.7	99.7	99.7	99.4	99.8

* Based on MP permeation.

**Table 3:** Comparison of different treatment technologies with MPs in liquid matrices.

Effluent	Technology	Mechanism	Efficiency (%)	Reference
Synthetic wastewater	Microfiltration	Retention	>99.0	This study
WWTP	Microfiltration	Retention	>72.0	[22]
WWTP	Microfiltration	Retention	<98.0	[23]
Laundry wastewater	Microfiltration	Retention	98.6	[21]
WWTP	Ultrafiltration	Retention	96.9	[20]
WWTP	Membrane bioreactor	Retention	79.0	[9]
WWTP	Coagulation/sedimentation	Retention	90.0	[7]
Laundry wastewater	Coagulation/sedimentation	Retention	98.0	[8]
WWTP	Electrocoagulation	Retention	90.0	[12]
Laundry wastewater	Electrocoagulation	Retention	98.0	[15]
Laundry wastewater	Microbubble flotation	Retention	98.0	[16]
WWTP	Photocatalysis	Degradation	44.7	[12]
Laundry wastewater	UVC/H_2_O_2_	Degradation	57.7	[11]

## Data Availability

The original data presented in the study are openly available in Zenodo at 10.5281/zenodo.15282048.

## Abbreviations

The following abbreviations are used in this manuscript:

COD	Chemical oxygen demand
MF	Microfiltration
MPs	Microplastics
PP	Polypropylene
PVDF	Polyvinylidene fluoride
UVC	Ultraviolet C
WWTP	Wastewater treatment plant

## References

[1] Monira S., Roychand R., Hai F.I., Bhuiyan M., Dhar B.R., Pramanik B.K. (2023). Nano and microplastics occurrence in wastewater treatment plants: A comprehensive understanding of microplastics fragmentation and their removal. Chemosphere.

[2] Ayankunle A.Y., Buhhalko N., Pachel K., Lember E., Kõrgmaa V., Mishra A., Lind K. (2023). Estimating Microplastics related to Laundry Wash and Personal Care Products released to Wastewater in Major Estonian Cities: A comparison of calculated and measured microplastics. J. Environ. Health Sci. Eng..

[3] Klinkhammer K., Kolbe S., Brandt S., Meyer J., Ratovo K., Bendt E., Rabe M. (2024). Release of fibrous microplastics from functional polyester garments through household washing. Front. Environ. Sci..

[4] Akter M.S., Chakraborty T.K., Ghosh G.C., Nice M.S., Zaman S., Khan A.S. (2024). Microplastics and heavy metals in freshwater fish species in the southwestern region of Bangladesh: An emerging concern for public health. Emerg. Contam..

[5] Li Y., Tao L., Wang Q., Wang F., Li G., Song M. (2023). Potential Health Impact of Microplastics: A Review of Environmental Distribution, Human Exposure, and Toxic Effects. Environ. Health.

[6] Thacharodi A., Hassan S., Meenatchi R., Bhat M.A., Hussain N., Arockiaraj J., Ngo H.H., Sharma A., Nguyen H.T., Pugazhendhi A. (2024). Mitigating microplastic pollution: A critical review on the effects, remediation, and utilization strategies of microplastics. J. Environ. Manag..

[7] Jachimowicz P., Cydzik-Kwiatkowska A. (2022). Coagulation and Flocculation before Primary Clarification as Efficient Solutions for Low-Density Microplastic Removal from Wastewater. Int. J. Environ. Res. Public Health.

[8] Li J., Dagnew M., Ray M.B. (2022). Effect of coagulation on microfibers in laundry wastewater. Environ. Res..

[9] Bayo J., López-Castellanos J., Olmos S. (2020). Membrane bioreactor and rapid sand filtration for the removal of microplastics in an urban wastewater treatment plant. Mar. Pollut. Bull..

[10] Koyuncuoğlu P., Erden G. (2021). Sampling, pre-treatment, and identification methods of microplastics in sewage sludge and their effects in agricultural soils: A review. Environ. Monit. Assess..

[11] Easton T., Koutsos V., Chatzisymeon E. (2023). Removal of polyester fibre microplastics from wastewater using a UV/H_2_O_2_ oxidation process. J. Environ. Chem. Eng..

[12] Xu Q., Huang Q.-S., Luo T.-Y., Wu R.-L., Wei W., Ni B.-J. (2021). Coagulation removal and photocatalytic degradation of microplastics in urban waters. Chem. Eng. J..

[13] Rayaroth M.P., Aravindakumar C.T., Shah N.S., Boczkaj G. (2022). Advanced oxidation processes (AOPs) based wastewater treatment—Unexpected nitration side reactions—A serious environmental issue: A review. Chem. Eng. J..

[14] Sturm M.T., Horn H., Schuhen K. (2021). Removal of Microplastics from Waters through Agglomeration-Fixation Using Organosilanes—Effects of Polymer Types, Water Composition and Temperature. Water.

[15] Akarsu C., Kumbur H., Kideys A.E. (2021). Removal of microplastics from wastewater through electrocoagulation-electroflotation and membrane filtration processes. Water Sci. Technol..

[16] Zhao H., Helgason A., Leng R., Chowdhury S., Clermont N., Dinh J., Aldebasi R., Zhang X., Gattrell M., Lockhart J. (2024). Removal of Microplastics/Microfibers and Detergents from Laundry Wastewater by Microbubble Flotation. ACS EST Water.

[17] Bu T., Mesa D., Pukkella A.K., Brito-Parada P.R. (2024). Optimising miniaturised hydrocyclones for enhanced separation of microplastics. Chem. Eng. J..

[18] Bule Možar K., Miloloža M., Martinjak V., Ujević Bošnjak M., Markić M., Bolanča T., Cvetnić M., Kučić Grgić D., Ukić Š. (2024). The Potential of AOP Pretreatment in the Biodegradation of PS and PVC Microplastics by Candida parapsilosis. Water.

[19] Dey T.K., Fan L., Bhuiyan M., Pramanik B.K. (2025). Evaluating the performance of the metal organic framework-based ultrafiltration membrane for nanoplastics removal. Sep. Purif. Technol..

[20] Tadsuwan K., Babel S. (2022). Microplastic abundance and removal via an ultrafiltration system coupled to a conventional municipal wastewater treatment plant in Thailand. J. Environ. Chem. Eng..

[21] Luogo B.D.P., Salim T., Zhang W., Hartmann N.B., Malpei F., Candelario V.M. (2022). Reuse of Water in Laundry Applications with Micro- and Ultrafiltration Ceramic Membrane. Membranes.

[22] Takeuchi H., Tanaka S., Koyuncu C.Z., Nakada N. (2023). Removal of microplastics in wastewater by ceramic microfiltration. J. Water Process Eng..

[23] Yahyanezhad N., Bardi M.J., Aminirad H. (2021). An evaluation of microplastics fate in the wastewater treatment plants: Frequency and removal of microplastics by microfiltration membrane. Water Pract. Technol..

[24] Pizzichetti A.R.P., Pablos C., Álvarez-Fernández C., Reynolds K., Stanley S., Marugán J. (2021). Evaluation of membranes performance for microplastic removal in a simple and low-cost filtration system. Case Stud. Chem. Environ. Eng..

[25] Hartinger M., Napiwotzki J., Schmid E.-M., Hoffmann D., Kurz F., Kulozik U. (2020). Influence of Spacer Design and Module Geometry on the Filtration Performance during Skim Milk Microfiltration with Flat Sheet and Spiral-Wound Membranes. Membranes.

[26] Ibrahim Y., Hilal N. (2023). Enhancing ultrafiltration membrane permeability and antifouling performance through surface patterning with features resembling feed spacers. NPJ Clean Water.

[27] Golgoli M., Khiadani M., Shafieian A., Sen T.K., Hartanto Y., Johns M.L., Zargar M. (2021). Microplastics fouling and interaction with polymeric membranes: A review. Chemosphere.

[28] Sturm M.T., Myers E., Schober D., Korzin A., Schuhen K. (2023). Development of an Inexpensive and Comparable Microplastic Detection Method Using Fluorescent Staining with Novel Nile Red Derivatives. Analytica.

[29] Tagg A.S., Harrison J.P., Ju-Nam Y., Sapp M., Bradley E.L., Sinclaird C.J., Ojeda J.J. (2017). Fenton’s reagent for the rapid and efficient isolation of microplastics from wastewater. Chem. Commun..

[30] Hurley R.R., Lusher A.L., Olsen M., Nizzetto L. (2018). Validation of a Method for Extracting Microplastics from Complex, Organic-Rich, Environmental Matrices. Environ. Sci. Technol..

[31] Li Q., Wu J., Zhao X., Gu X., Ji R. (2019). Separation and identification of microplastics from soil and sewage sludge. Environ. Pollut..

[32] Ormaniec P., Mikosz O.J. (2022). A review of methods for the isolation of microplastics in municipal wastewater treatment. Tech. Trans..

[33] Tong H., Jiang Q., Zhong X., Hu X. (2021). Rhodamine B dye staining for visualizing microplastics in laboratory-based studies. Environ. Sci. Pollut. Res..

[34] Tse Y.-T., Lo H.-S., Tsang C.-W., Han J., Fang J.K.-H., Chan S.M.-N., Sze E.T.-P. (2023). Quantitative analysis and risk assessment to full-size microplastics pollution in the coastal marine waters of Hong Kong. Sci. Total Environ..

[35] Pistocchi A., Aloe A., Dorati C., Alcalde Sanz L., Bouraoui F., Gawlik B., Grizzetti B., Pastori M., Vigiak O. (2017). The Potential of Water Reuse for Agricultural Irrigation in the EU: A Hydro Economic Analysis.

[36] Park H.-G., Cho S.-G., Kim K.-J., Kwon Y.-N. (2016). Effect of feed spacer thickness on the fouling behavior in reverse osmosis process—A pilot scale study. Desalination.

[37] Nasir M.S., Tahir I., Ali A., Ayub I., Nasir A., Abbas N., Sajjad U., Hamid K. (2024). Innovative technologies for removal of micro plastic: A review of recent advances. Heliyon.

